# New software

**DOI:** 10.1155/S1463924602000202

**Published:** 2002


Journal of Automated Methods & Management in Chemistry
Vol. 24, No. 6 (November–December 2002) pp. 209–210

Taylor & Francis

Taylor & Francis Group

### New software

### Fast new crystal data import and moving models enhance INCA Crystal

A fast new method for viewing, importing and editing crystal structures, and the dynamic display of crystal orientation during data acquisition are among the significant benefits now available to users of the INCA microanalysis suite from Oxford Instruments Analytical.

Analytical's PC-based INCA platform is already very highly regarded for its powerful high-accuracy analysis capability and ease of use. New features in the latest issue software (issue 12) offer both new functionality and a number of usability improvements.

Users of INCA Crystal, which offers superb texture and grain boundary analysis by electron backscattered diffraction (EBSD), can now review and import all the crystallographic parameters needed to perform analysis on a given sample, quickly and simply. The new View Phase in INCA Crystal's navigation system directly accesses industry standard '.CIF' crystal structures, which can be used without conversion, and also provides the full range of symmetry information, i.e. space group, etc. for information. The new Edit Phase step allows the import or entry of new structures, either from a database or from printed material, with great ease and flexibility. Both new steps display crystal structures with a highly detailed Crystal Model or 'Mimic'.

This latest version of INCA Crystal also features enhanced colour contour plotting in pole figures and inverse pole figures. Users can, according to preference, position the RD axis at either the top or the side of pole figures. More expert users can also now opt for either equal area or stereographic pole figure plotting methods.

Moving pictures during data collection are also available for those who like to keep one eye on crystal orientation as they acquire data. A Crystal Mimic is now constantly updated in 'real time', so orientation can be viewed as a dynamically changing image—extremely informative in the analysis of deformed materials. The Crystal Mimic is also available in texture analysis—just click on a point and see the orientation.

*For further information, contact Analytical, Halifax Road, High Wycombe HP12 3SE, UK. Tel: +44 (0)1494 442255; Fax: +44 (0)1494 461033; e-mail: analytical@oxinst.co.uk. Internet: http://www.oxford-instruments.com*

### Zymark introduces new system integration software

Zymark Corporation announces the release of CLARA™ 2.2, the third iteration of the CLARA software suite, which continues to build upon its industry leading performance and ease of use. Developed for the scientists' needs, CLARA is an integral part of any successful drug-discovery automation solution.

CLARA incorporates new Plug-and-Play resource adapters to provide a simplified environmental set-up. Any instrument in the automated system is instantly identified by CLARA and ready for drag-and-drop assay creation. CLARA has more than 100 adapters for third-party instruments, giving users an unprecedented degree of system flexibility. In addition, its optimization tools have been enhanced further to improve assay automation and throughput. The well-known application scheduler has been updated increasing computational speed. The new real-time Gantt Chart displays run-time progress and completion of instructions, enabling effective resource utilization.

Assay development and validation is streamlined by one-click simulation of any instrument in the system. Advanced User Notification allows assays to run unattended: cell phones, pagers and e-mail accounts can be contacted in response to resource errors, data milestones or quality assurance statistics.

Available for use with every Zymark Twister® II and integrated system, CLARA 2.2 is available as a new or upgrade package. The software is key to the development of HTS, genomic and proteomic automated solutions.

*For more information, contact Lynda Thomas. Tel: +1 508 497-6549; e-mail: lynda.thomas@zymark.com; Internet: http// www.zymark.com*

### News

### VelQuest partners with Waters

VelQuest and Waters Corporation have announced a marketing partnership and joint effort to provide a seamless interface to the Waters Millennium$^{32}$® Chromatography Data System (CDS) to help scientists manage scientific data better. First introduced in 1992, Millennium$^{32}$ was the first of its kind to incorporate fully an Oracle® relational database allowing chemists to locate information effortlessly and spot trends in their chromatographic analyses. The Millennium$^{32}$ Interface will allow VelQuest's electronic notebook (ePMC) sys-

